# The InP(100) Surface Phase Diagram: From the Gas Phase to the Electrochemical Environment

**DOI:** 10.1021/acsami.4c20370

**Published:** 2025-01-21

**Authors:** Holger Euchner, Vibhav Yadav, Matthias M. May

**Affiliations:** Universität Tübingen, Institute of Physical and Theoretical Chemistry, Auf der Morgenstelle 15, 72076 Tübingen, Germany

**Keywords:** InP(100), Surface Reconstruction, Phase Diagram, Computational Hydrogen Electrode, Density Functional Theory

## Abstract

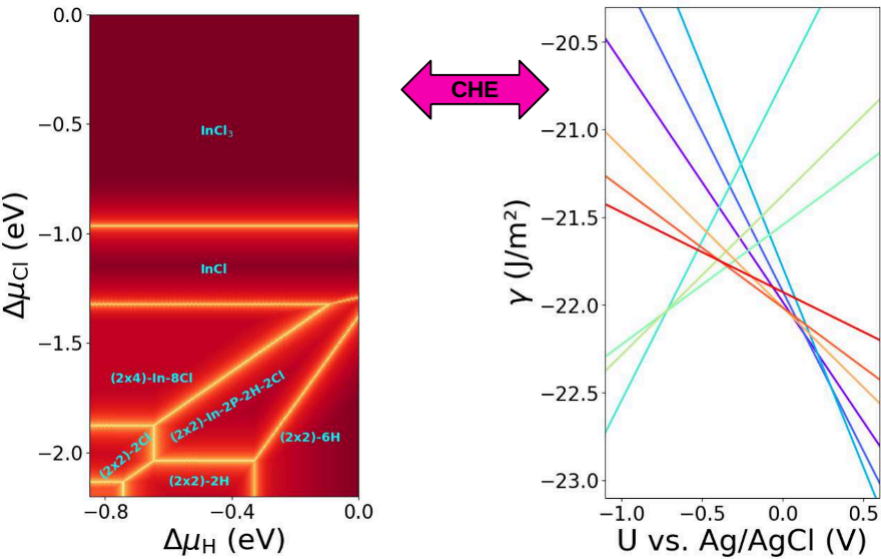

The versatile optoelectronic properties of the material class of III–V semiconductors enable the highest performance in photovoltaic and photoelectrochemical solar cells. While a high level of control and understanding with respect to different surface reconstructions of these compounds in gas-phase ambient has been reached, the situation in an electrochemical environment still poses challenges. Here, we therefore have undertaken a computational study of the InP(100) surface in the presence of hydrogen and chlorine, mimicking the contact with a hydrochloric acid-containing electrolyte, aiming at an understanding of ion adsorption and dominant surface reconstructions with respect to applied potential and electrolyte concentration. For this purpose, the most stable surface terminations for hydrogen and chlorine (co)adsorption from the gas phase as well as the corresponding phase diagrams have been determined with respect to the hydrogen and chlorine chemical potential. In this context, we also introduce a quantitative type of phase diagram to highlight the stability of surface phases with respect to competing structures. Finally, by making use of the computational hydrogen electrode approach, these results were then transferred to the potential domain. We find that hydrogen (chlorine) adsorption is dominating at more (less) cathodic potentials, while coadsorption is limited to small fractions of the phase space. This allows us to determine experimentally accessible phases for which no detrimental effects, such as the creation of in-gap surface states, are to be expected.

## Introduction

The transformation toward a defossilized energy supply of our society has to be realized in the near future to mitigate global warming, meaning that not only electricity but also fuels have to be replaced with renewable alternatives. Here, the production of hydrogen will be part of the solution, with photoelectrochemical (PEC) water splitting having the potential to become an important building block for the future distributed production of carbon-neutral fuels.^1−3^ The PEC method is based on an integrated approach, where the photoelectrode is directly immersed in the electrolyte such that photon harvesting and water splitting happen in a single device.^4,5^ This comes along with advantages such as compact device design, independence from the electricity grid, and co-benefits from heat exchange between a solar absorber and catalyst.^6^ However, the direct contact of the solar absorber and electrolyte results in numerous challenges, in particular with respect to the electrochemical interface. Similar to the case of batteries, the electrochemical interface of the electrode and electrolyte is crucial for the understanding and improvement of the underlying processes, especially with respect to device stability.

Photoelectrodes applied for direct solar water splitting typically show severe stability issues in the electrochemical environment, which directly leads either to dissolution of the electrode or to the degradation-induced formation of surface states in the band gap of the photoabsorber, causing charge-carrier recombination and performance loss. While the successful passivation/functionalization of InP-based photocathodes recently allowed for largely improved performance, the exact structure of the stable interfaces and its functioning are still an open question.^7^ In a first approach, we therefore study the simple binary InP(100) surface—*the drosophila* of III–V photoelectrochemistry—to gain insight into the complex structure and processes occurring at the electrode/electrolyte interface.

For this purpose, we have studied the surface phase diagram of InP in an electrochemical environment, consisting of aqueous solutions of hydrochloric acid (HCl), via density functional theory (DFT). Here, it has to be noted that studies considering the phase stability beyond the clean InP(100) surface are rather limited,^8−14^ while electrochemical environments, including the impact of electrolyte constituents and applied potential on the surface structure, so far have not been addressed at all. Hence, starting from different surface reconstructions of InP(100), we have investigated the adsorption of hydrogen and chlorine upon the latter ones, motivated by the finding that highly ordered interfaces/phases exist under these conditions, whose exact structures are, however, not yet fully identified.^15^ The resulting surface phase diagrams, obtained for hydrogen and chlorine adsorption in a vacuum (from the gas phase) and depicted as a function of the respective chemical potentials (μ_H_ and μ_Cl_), contain a variety of stable phases that show hydrogen and chlorine adsorption. However, only for a small part of the phase space, i.e., for very specific conditions, is the coadsorption of hydrogen and chlorine observed. Furthermore, to translate these findings to an electrochemical environment, the concept of the computational hydrogen electrode has been applied.^16^ This consequently allows the determination of the surface phase diagram in an aqueous electrolyte, by evaluating the surface free energy as a function of the electrochemical potentials of hydrogen  and chlorine . Finally, by selecting a certain electrolyte composition, the phase diagram can be transformed in the potential domain, such that the phase stability with respect to an applied potential can be extracted, in principle allowing for comparison to the corresponding experimental data.^15,17^

## Methods

Density functional theory-based calculations have been performed to determine the surface phase diagram of InP in the presence of hydrogen and chlorine. For this purpose, different, well-known reconstructions of the pristine InP(100) surface, as can be prepared in ambient gas-phase conditions,^18^ have been optimized and used as starting configurations for hydrogen and chlorine adsorption. To allow for an efficient modeling of the respective surfaces, asymmetric slabs with pseudohydrogen termination at the bottom were constructed, thus preventing the occurrence of dangling bonds and spurious states in the band structure. All DFT calculations were conducted with the CP2K code using the Gaussian and plane wave (GPW) method, using the DZVP-MOLOPT-SR-GTH basis set in combination with the Godecker–Teter–Hutter pseudopotentials, applying a cutoff of 800 Ry together with a relative cutoff of 60 Ry.^19,20^ Exchange and correlation were described via the PBE functional, while in addition, a dispersion correction was applied as introduced by Grimme et al. (vdW-D3).^21,22^ First, the bulk structure of InP was optimized with the above-described settings, yielding a lattice parameter of 5.935 Å for the cubic unit cell. Starting from this structure, different surface unit cells for the InP(100) surface were constructed. All investigated surfaces were based on a 12-layer structure (six In and six P layers) with fixed cell dimensions along the *c*-axis, such that each structure was terminated by a vacuum layer of at least 15 Å thickness (see Figure S1 in the Supporting Information (SI)).

## Computational Hydrogen Electrode

To access surface phase diagrams in the gas-phase ambient, a grand canonical approach has to be applied,^23,24^ relating the calculated surface energies to different experimental conditions, corresponding to the availability of the respective atoms. This can be achieved by expressing the Gibbs free surface energy γ(*T*, *p*) as a function of the chemical potential, μ_*i*_, of the corresponding gas-phase molecules, normalized to the surface area *A*. This results in the following equation:
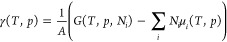
1

Here, *N*_*i*_ is the number of atoms of type *i*, whereas *G* is the Gibb’s free energy of the slab, which in ab initio-based approaches is frequently approximated by the corresponding total energy *E*_slab_, meaning that entropy and volume change are neglected. By introducing Δμ_*i*_(*T*, *p*) = μ_*i*_(*T*, *p*) – *E*_*i*_, the chemical potential is typically renormalized with respect to the ground state energy of the respective elements *E*_*i*_, thus yielding
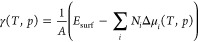
2with *E*_surf_ = *E*_slab_ – *∑*_*i*_*N*_*i*_*E*_*i*_. Starting from this equation, the most stable surface structures, those with the lowest surface free energy, can be determined.

When systems in the electrochemical environment are considered, the presence of charged species has to be accounted for, which means that, in the above formalism, the chemical potential has to be replaced by the electrochemical potential . Accessing the surface free energies in the electrochemical environment, on the other hand, would in principle mean calculating solvation free energies for the respective ions in solution. This is, however, a computationally extremely demanding task. Fortunately, there exists an elegant and well-established approach to connect the electrochemical potentials of molecules in the gas phase and their corresponding ions in solution: the computational hydrogen electrode (CHE).^16^ The underlying idea of the CHE is the fact that the standard hydrogen electrode (SHE) at standard conditions yields a reference point at which hydrogen gas and protons in solution are in equilibrium, i.e., . Consequently, by exploiting this relation, one only has to compute the energy of a hydrogen molecule in the gas phase, instead of directly computing the corresponding solvation energies.^16,24,25^ Since the potential dependence of an electron at the Fermi level and the pH dependence of an ion in solution are well-known, the electrochemical potential of a proton and electron in solution can be expressed by the following equation:
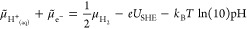
3with *U*_SHE_ the potential with respect to the standard hydrogen electrode scale. It is now easy to extend this concept to any other species in solution.^26,27^ For the case of chlorine the corresponding equation reads as
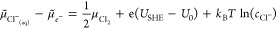
4with *U*_0_ the standard potential of the Cl/Cl^–^ redox couple (i.e., 1.36 V) versus SHE.^28^ Note that in eq 4 the concentration is used instead of the formally correct activity, an approximation valid for low concentrations.^29^

In the here-described grand canonical approach, the temperature-, concentration-, and potential-dependence of the electrochemical potential can also be renormalized and combined in a single term, Δμ̃, yielding the following equation for the case of protons:
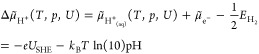
5

For the chlorine ion, one obtains
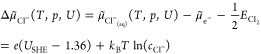
6

In this formulation, the total energy of the gas-phase molecules is subtracted from the electrochemical potential of the solvated ion and corresponding electrons. This renormalization is, as in the above-discussed vacuum case, based on the assumption that the free energies of the molecules can be approximated by the total energy that is directly accessible by DFT. Making use of the above derived expressions, the change in Gibbs free surface energy can finally be expressed as
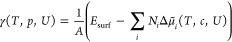
7

This now enables us to also compute phase diagrams as a function of the electrochemical variables. However, it must be pointed out that the typical application of the CHE, as outlined above, comes with certain limitations. The fact that in eq 7 total energies are used means that the electrochemical environment is not directly accounted for as the free energy is assumed to be independent of the electrochemical variables such as the applied potential.^30^ Furthermore, in the methodology applied here, where DFT calculations in a vacuum are considered, effects such as the charging of the electrode and the electrochemical double layer and their effect on energetics and electronic structure (band bending) are not accounted for. Consequently, results for semiconducting systems in contact with an electrolyte obtained by the CHE approach are strictly speaking valid for only uncharged interfaces. Unlike for metals, a surface charge on a semiconductor will not be fully screened, leading to a finite electric field within the semiconducting slab.^31,32^ This will effectively introduce a certain level of uncertainty with respect to the potential domain when comparing these results directly with experiment.

## Results and Discussion

In the following, the InP(100) surface and its reconstructions are addressed in a stepwise approach. Starting from the clean surface in the presence of only In and P, the adsorption of hydrogen and chlorine is considered separately before a combined adsorption is investigated. These scenarios are first considered for gas-phase ambient conditions before the results are exploited for a given electrochemical environment.

### Clean Surface

First, the phase diagram of the pristine InP(100) surface (in vacuum) has been reinvestigated with respect to the chemical potential of In and P, which in experiment are determined by the exact growth/surface preparation conditions. Since the chemical potentials of In and P are interrelated via the stability of the InP bulk phase under equilibrium conditions (μ_InP_ = μ_In_ + μ_P_), the surface energy can be expressed as a function of only the In chemical potential:
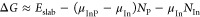
8

The resulting phase diagram shows the so-called (2 × 2)-2D, *c*(4 × 4), α2(2 × 4), β2(2 × 4), and mixed-dimer phases as stable clean surfaces. Since all further calculations are based on the surface reconstructions introduced here, these will be described in some detail in the following. The (2 × 2)-2D surface corresponds to a P-terminated surface with two additional P-dimers on top. The *c*(4 × 4) phase is closely related to the (2 × 2)-2D reconstruction, being constructed from a larger supercell that is also terminated by phosphorus with three additional P-dimers on top. Finally, there are three different structures that are based on 2 × 4 supercells. The α2(2 × 4) and β2(2 × 4) phases can be understood as stepped surfaces, which differ in the number of terminating P atoms. The mixed dimer structure, on the other hand, corresponds to an In-terminated surface with a single, “mixed” In–P dimer on top.

The phase diagram shows that with increasing In chemical potential, the more In-rich surface terminations become dominant, culminating in the mixed dimer surface. For intermediate Δμ_In_ values, the α2(2 × 4) and β2(2 × 4) phases are the most stable ones. When Δμ_In_ decreases further, the *c*(4 × 4) is observed for a small potential range. Finally, for P-rich conditions, the (2 × 2)-2D surface is stabilized. Apart from the fact that the (2 × 2)-1D structure is found to be slightly unstable, this is in agreement with literature.^33^ These differences are likely due to the different exchange–correlation functionals and the negligence of van der Waals interactions in earlier calculations.

**Figure 1:**
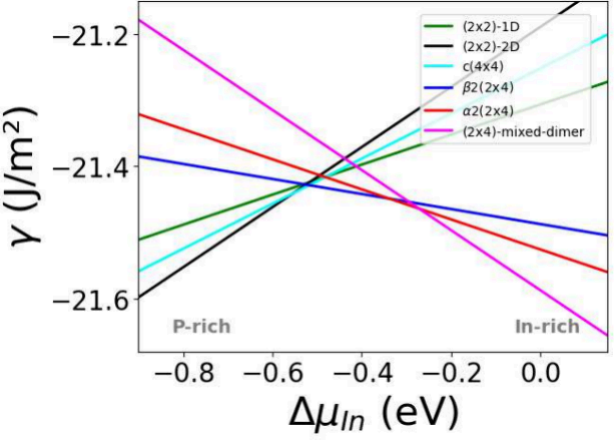
Phase diagram of the clean InP(100) surface as a function of the In chemical potential. The corresponding structures are depicted in Figure 2.

**Figure 2:**
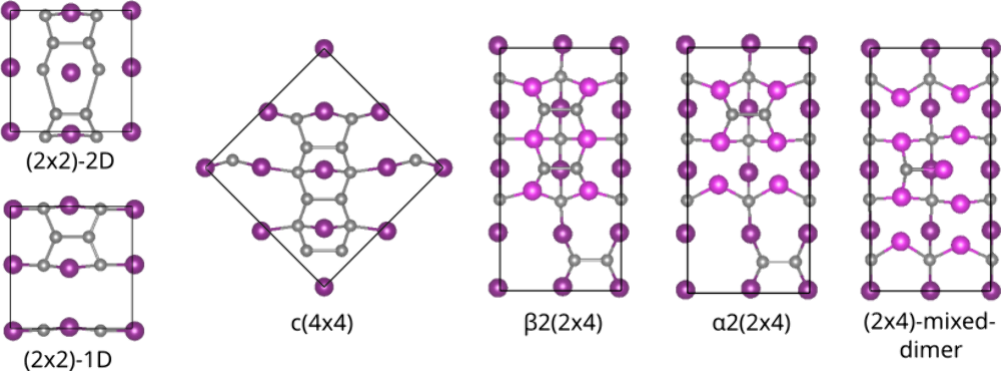
Different surface reconstructions of the InP(100) surface. In atoms are depicted in purple (and pink if two In layers are shown), while P atoms are shown in gray.

### Hydrogen and Chlorine Adsorption

**Figure 3:**
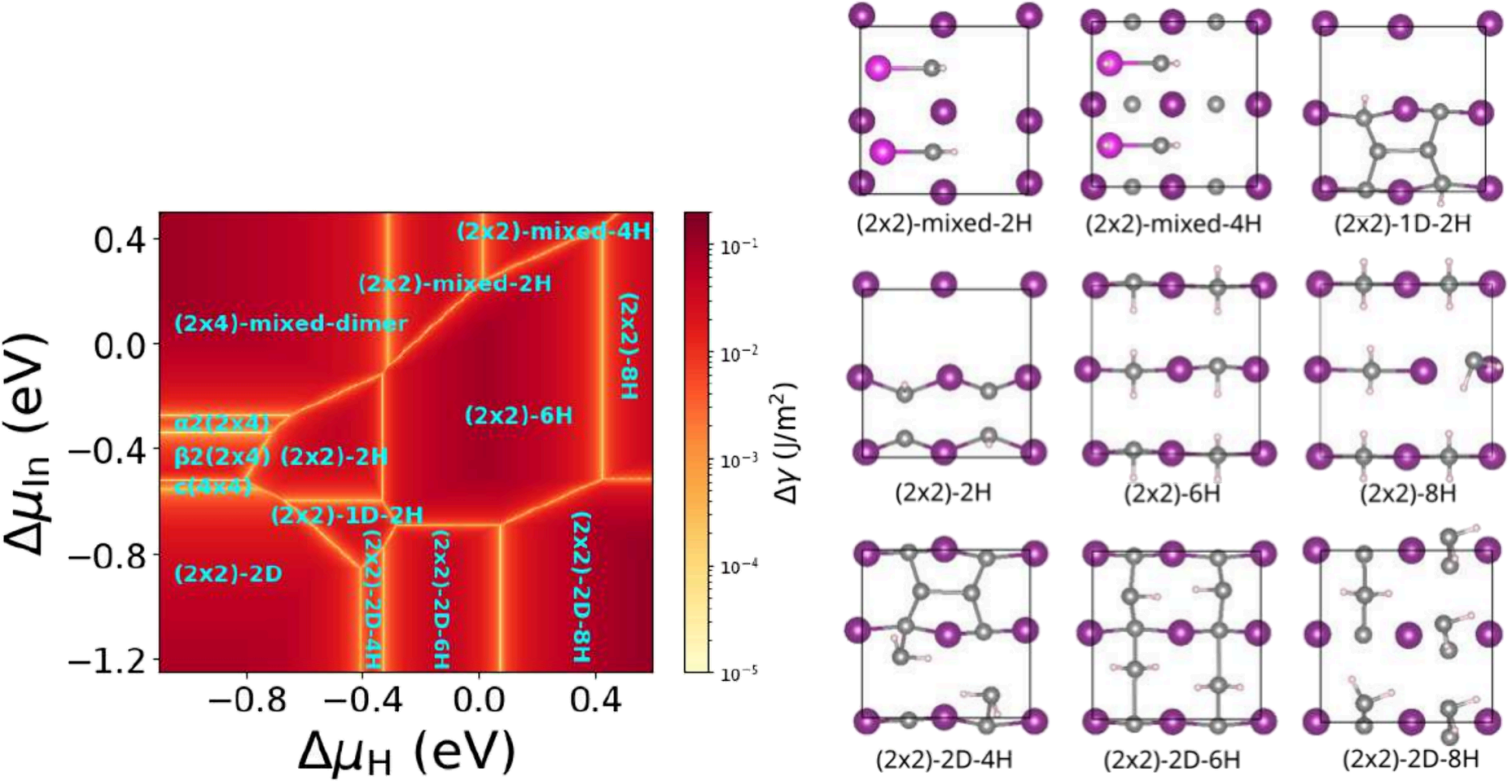
Phase diagram of the InP(100) surface with respect to the In and H chemical potential and corresponding structures (top view). In and P atoms of the topmost layers are depicted in purple (and pink if two In layers are shown) and gray, whereas H is shown in light pink.

As the next step, the impact of hydrogen was investigated by computing the corresponding phase diagram in the presence of hydrogen. For this purpose, the different stable plain surfaces and derivatives thereof were decorated with different numbers and arrangements of hydrogen atoms. In total more than 300 different configurations were structurally optimized, finally yielding the corresponding surface energies as a function of the renormalized In and hydrogen chemical potentials Δμ_In_ and Δμ_H_ as depicted in Figure 3. Before the discussion of the resulting phase diagram, a consistent nomenclature for hydrogen-terminated surfaces will be introduced. In the following, structures with hydrogen on P-terminated (2 × 2) surfaces will be termed (2 × 2)-*n*H phases, whereas phases based on the P-dimer terminated (2 × 2)-2D phase will be termed (2 × 2)-2D-*n*H phases. Here, a note of caution is necessary, as in earlier works a (2 × 2)-2D-2H structure is reported that refers to the P-terminated surface without additional phosphorus,^18^ which in the here introduced convention corresponds to the (2 × 2)-2H phase.

Regarding the phase diagram, as expected, the pristine surface reconstructions are observed at low Δμ_H_ values (low Δμ_H_ corresponds to a low hydrogen partial pressure). For increasing Δμ_H_, hydrogen-terminated surfaces become stabilized. Interestingly, under P-rich conditions as well as In-rich conditions, the pristine clean phases remain stable over a wider range of Δμ_H_. Here, it has to be noted that the phase diagram differs somewhat from literature,^18^ which again may be attributed to the use of different exchange–correlation functionals and/or the larger number of competing structures that were considered in our study. Furthermore, it must be pointed out that the energy differences between certain configurations are often rather small.

To take these uncertainties into account, the phase diagram is represented such that the stability of the thermodynamically stable phases with respect to competing phases of different stoichiometry is color-coded. This is achieved by computing the energy difference between the most stable and second most stable phases at a particular value of Δμ_H_ and Δμ_In_. This value is then used as a descriptor for phase stability and represented by the color code in Figure 3. Hence, the darker a particular area is depicted in the phase diagram, the likelier the occurrence of the corresponding phase. A large part of the phase diagram comprises the (2 × 2)-*n*H phases (with *n* = 2, 6, 8), which correspond to phosphorus-terminated surfaces with hydrogen atoms replacing the top P-dimer. Here, the (2 × 2)-6H phase covers a rather large area and is, as can be inferred from the darker color, also expected to be quite stable. The maximum coverage is reached when each top layer phosphorus atom is terminated by two hydrogen atoms. The resulting (2 × 2)-8H phase already shows distortions of the PH_2_ units at the surface and is only stable for Δμ_H_ > 0.4. For P-rich conditions, (2 × 2)-2D-based structures with hydrogen atoms filling the available sites on the P-dimers become stable for increasing Δμ_H_. Here, again, the maximum hydrogen content on the surface seems to be reached with a total of 8 hydrogen atoms, meaning a P-terminated surface with 4 PH_2_-units on top. Finally, for In-rich conditions and increasing Δμ_H_, mixed-dimer-like surface terminations are found to be the most stable. The (2 × 2)-mixed phases correspond to an In-rich surface that is terminated by two mixed dimers, where first both P-sites ((2 × 2)-mixed-2H) and then both In-sites ((2 × 2)-mixed-4H) of the In–P dimers are occupied by hydrogen. Interestingly, the α2(2 × 4)- and β2(2 × 4)-based phases are not observed any more when hydrogen is present. This is because for intermediate Δμ_In_ values the fully P-terminated (2 × 2)-based phases are stabilized. This stabilization is a consequence of the higher availability of P-sites for the formation of P–H bonds. Here, it has to be noted that not all of the structures that are found to be stable—in particular those based on the P-rich (2 × 2)-2D surface—comply with the electron counting rules introduced by Pashley.^34^ Finally, it has to be pointed out that, while a large number of different configurations have been investigated, it cannot be excluded that larger supercells may yield additional stable reconstructions with intermediate hydrogen content.

**Figure 4:**
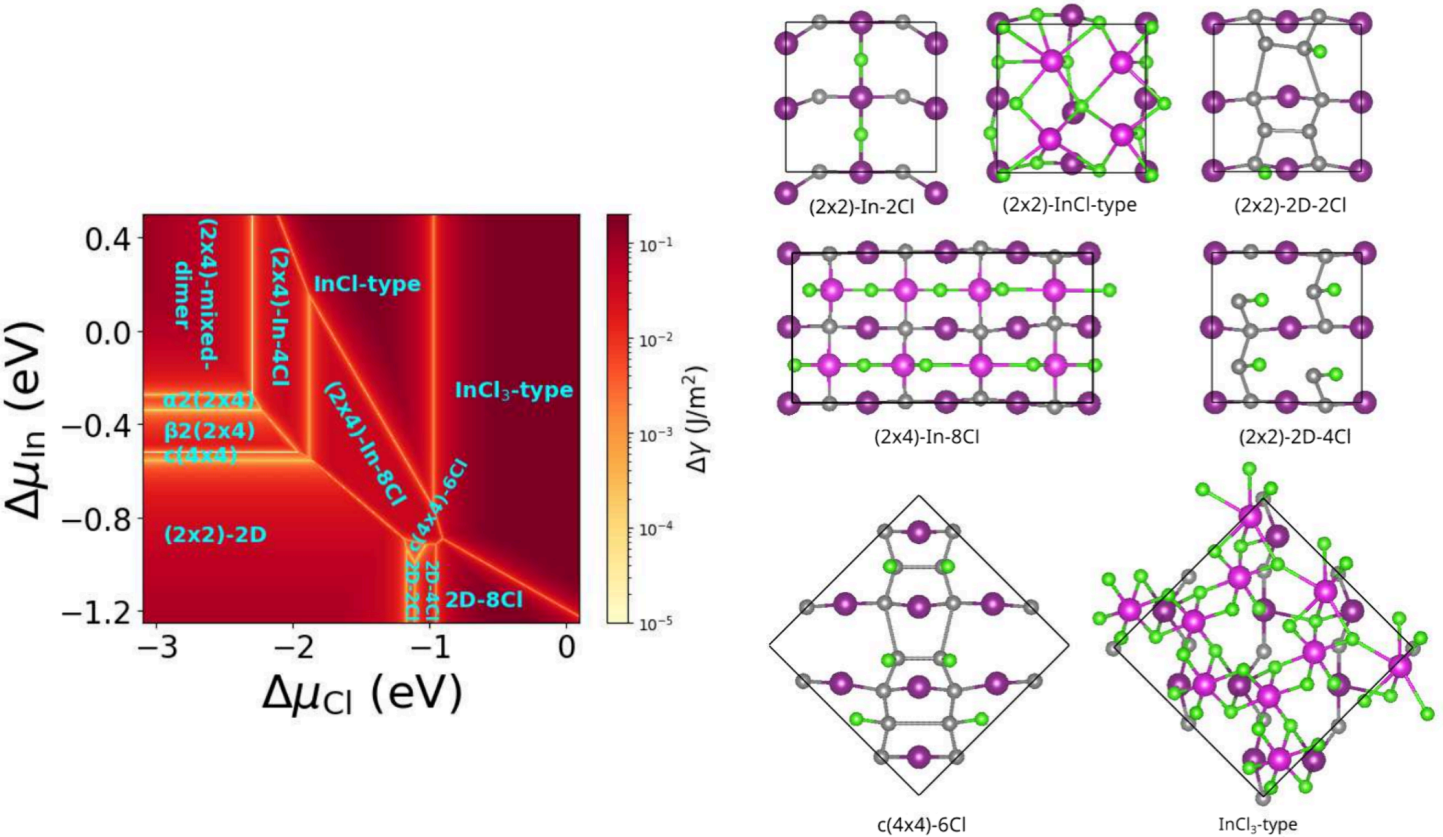
Phase diagram of the InP(100) surface with respect to the In and Cl chemical potential and corresponding structures (top view). In and P atoms of the topmost layers are depicted in purple (and pink if two In layers are shown) and gray, whereas chlorine is shown in light green.

Similarly, the adsorption of chlorine on the different InP(100) surface reconstructions was considered, now allowing us to compute the phase diagram as a function of the indium and chlorine chemical potentials Δμ_In_ and Δμ_Cl_, as shown in Figure 4. Again, the pristine surface reconstructions are observed at low Δμ_Cl_ values. Here, it has to be noted that they are observable for significantly lower chemical potentials, as in the case of hydrogen, which is a consequence of the higher reactivity of chlorine. For increasing Δμ_Cl_, chlorine-terminated surfaces become quickly stabilized. For increasing Δμ_Cl_ and under P-rich conditions, the pristine (2 × 2)-2D phase shows an extended stability range and is followed by surfaces for which an increasing number of Cl atoms are located on top of the P-dimer sites, with the (2 × 2)-2D-8Cl phase already being largely disordered. For In-rich conditions, the In-terminated (2 × 2)-In-2Cl and (2 × 4)-In-8Cl phases become stable for increasing Δμ_Cl_ values, before an InCl-like layer becomes stabilized. Interestingly, at a large Δμ_Cl_, the formation of an InCl_3_-like layer becomes stable over the whole Δμ_In_ range. Both InCl-type phases cover a rather large range of the phase space and are, as can be seen from the dark color in the phase diagram, expected to be comparably stable. As can be inferred from the large part of the phase space that is covered by In-rich surface terminations, chlorine has a strong preference to form In–Cl bonds, whereas P–Cl bonds only form under P-rich conditions. As in the case of hydrogen, there may exist larger supercells that stabilize additional intermediate Cl concentrations.

Following the same approach as in the previous paragraphs, the co-adsorption of hydrogen and chlorine was considered. Now, the different plain surfaces and their derivatives were decorated by different numbers and arrangements of hydrogen and chlorine atoms. In principle, the phase diagram can now be obtained as a function of the renormalized chemical potentials of In, Cl, and H. However, to allow for a better graphical representation, phase diagrams for three selected values of the In chemical potential have been chosen. The resulting graphs correspond to two-dimensional cuts through the three-dimensional phase space, spanned by the chemical potentials of In, Cl, and H. Choosing a fixed value for Δμ_In_ can also be understood as chlorination/hydrogenation of a particular surface reconstruction. The resulting phase diagrams for In-rich (Δμ_In_ = −0.9 eV), intermediate (Δμ_In_ = −0.4 eV), and P-rich (Δμ_In_ = 0.1 eV) conditions correspond to selecting the (2 × 2)-2D, β2(2 × 4), and the mixed-dimer reconstructions as clean surfaces, respectively.

**Figure 5:**
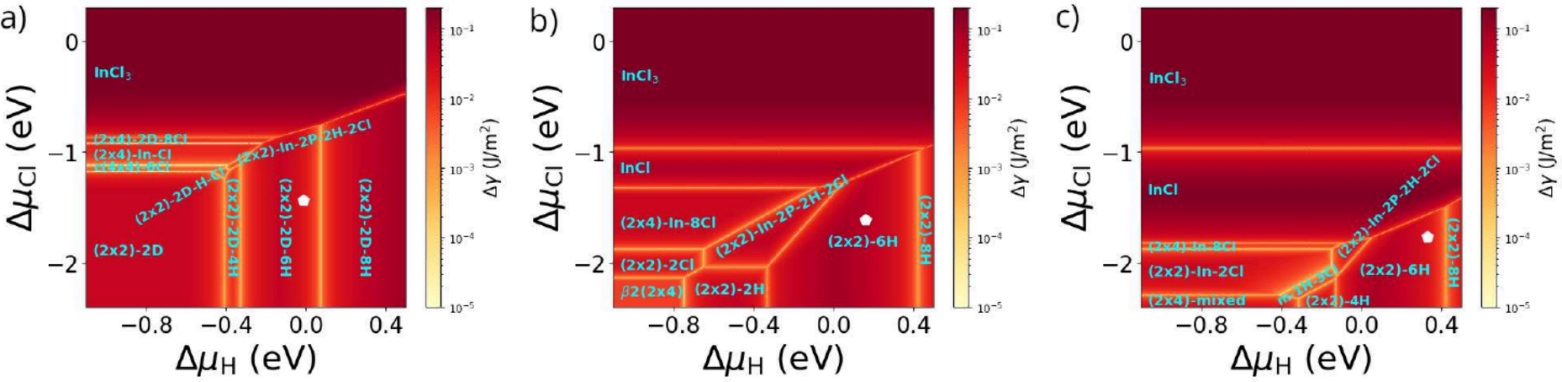
Phase diagram of the InP(100) surface with respect to the chemical potentials of H and Cl for a fixed In chemical potential. (a) In-rich (Δμ_*In*_ = −0.9 eV), (b) intermediate (Δμ_In_ = −0.4 eV), and (c) P-rich (Δμ_In_ = 0.1 eV) conditions. The white pentagons represent particular combinations of Δμ_In_, Δμ_H_, and Δμ_Cl_, corresponding to applied potentials of approximately −0.25, −0.42, and −0.58 V, respectively.

These combined phase diagrams, depicted in Figure 5a–c, clearly show the dominant nature of chlorine adsorption. For increased chlorine chemical potentials, the InCl_3_-like overlayer is observed for the different Δμ_In_ values and, moreover, is essentially independent from the hydrogen chemical potential. The dark color of the corresponding phase space area indicates that InCl_3_-type phases are significantly more stable than the competing structures that have been investigated. Only at low chlorine chemical potential are hydrogen-containing phases observed. For more In-rich conditions (Figure 5b and c), the phase space that is dominated by chlorine-rich phases is further increasing, while hydrogen-containing surfaces are only observed for further decreased Δμ_Cl_ values. This essentially confirms our previous findings for the separate hydrogen and chlorine absorption and can be understood from the fact that hydrogen prefers to bind to phosphorus, whereas Cl prefers to form bonds with In. Consequently, the more indium-rich the conditions are, the more dominant the Cl adsorption and hence the Cl-rich phases. However, even under P-rich conditions, chlorine adsorption takes place for a large part of the phase diagram.

**Figure 6:**
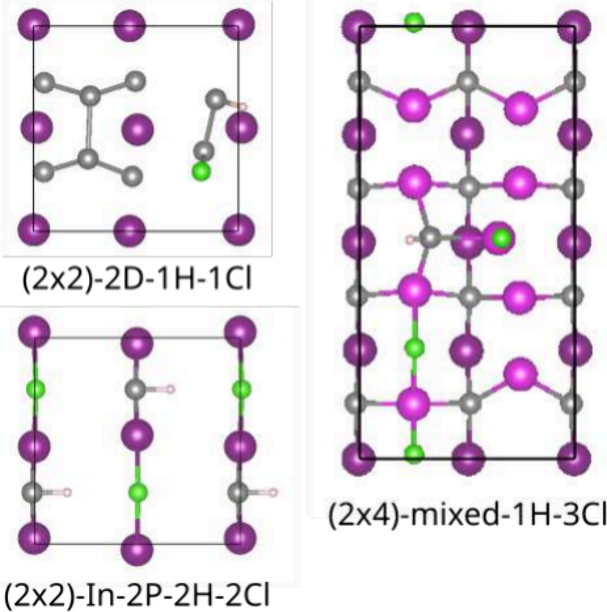
Surface reconstructions with hydrogen and chlorine co-adsorption (top view). In and P atoms of the topmost layers are depicted in purple (and pink if two In layers are shown) and gray, whereas chlorine and hydrogen are shown in light green and light pink.

Interestingly, for the three selected In chemical potentials, the co-adsorption of hydrogen and chlorine is only observed for a very narrow range in chemical potential. This corresponds well with adsorption studies on metal electrodes,^26^ where similar behavior was observed. In the case of P-rich conditions (Δμ_In_ = −0.9 eV), two tiny areas with hydrogen/chlorine co-adsorption are observed (see Figure 5a). For rather low values of Δμ_Cl_ and Δμ_H_, a (2 × 2)-2D-based structure with a hydrogen and a chlorine atom sitting on the same dimer, the (2 × 2)-2D-H-Cl phase, is observed (see Figure 6). Increasing Δμ_Cl_ and Δμ_H_ values foster an In-terminated surface with partial P-coverage, the (2 × 2)-In-2P-2H-2Cl structure, with the P atoms being saturated by hydrogen and Cl atoms occupying the free P-sites. At larger In chemical potential (Δμ_In_ = −0.4 eV), only the latter co-adsorption phase prevails, which is a consequence of its increased In content as compared to the (2 × 2)-2D-H-Cl phase. In fact, the (2 × 2)-In-2P-2H-2Cl phase even occupies an increased area in the phase diagram, as can be seen in Figure 5b. Finally for Δμ_In_ = 0.1 eV, again the (2 × 2)-In-2P-2H-2Cl phase is observed, as well as a mixed dimer based surface (mixed-1H-3Cl) with the P and In atoms of the dimer being saturated by H and Cl and two additional Cl atoms forming In–Cl–In bonds (see Figure 6).

### From Gas-Phase to Electrochemical Environment

While the phase diagrams discussed in the previous paragraphs are relevant for the adsorption of hydrogen and chlorine in a gas-phase environment, which is of great importance for epitaxial growth, the question of the predominant phases for systems in contact with an electrochemical environment remains open. However, making use of the above-introduced CHE concept and also applying it for the case of indium allow the transfer of the obtained phase diagrams to the potential domain. This means the phase diagram can be expressed with respect to the applied potential for given concentrations of hydrogen, chlorine, and indium in the electrolyte solution. For this purpose, the standard potentials of the corresponding redox couples have to be used to relate them to the SHE scale (see Table 1).

**Table 1:** Redox Potentials of H/H^+^, In/In^3+^, Cl/Cl^–^, and Ag/AgCl vs SHE

redox couple	potential (vs SHE)
H/H^+^	0 V
In/In^3+^	–0.338 V^28^
Cl/Cl^–^	+1.360 V^28^
Ag/AgCl	–0.197 V^35^

**Figure 7:**
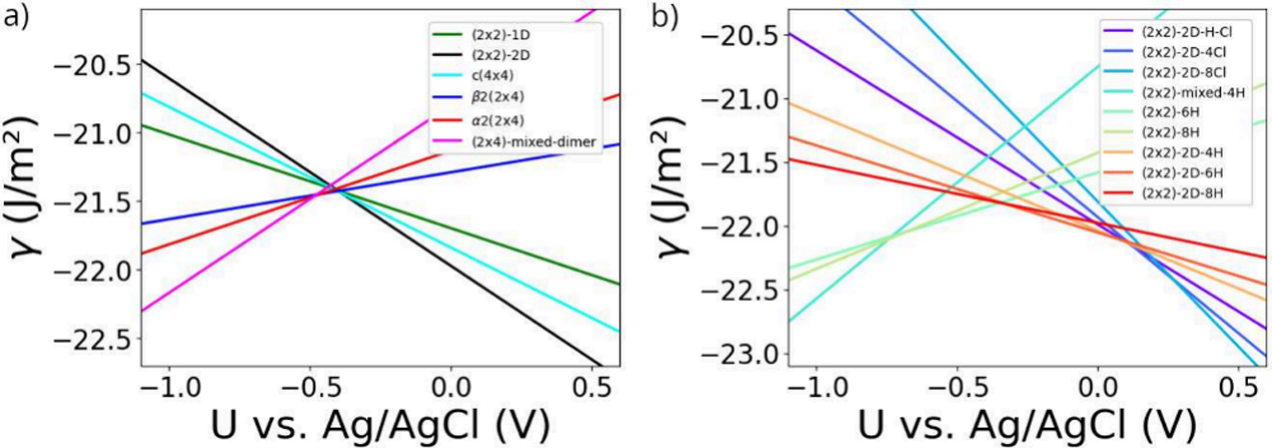
Phase diagram of the InP(100) surface as a function of the applied potential for a 0.01 molar InCl_3_ solution at pH 1. In (a) only the stability of the plain surface reconstructions is considered, while (b) considers H and Cl adsorption.

Consequently, for fixed In, Cl, and H concentrations, as in an electrochemical experiment, the phase diagram can be depicted as a function of the applied potential. In the following, the concentrations of In, Cl, and H are assumed to stem from the dissolution of 0.01 molar InCl_3_ in 0.1 molar hydrochloric acid (pH 1), thus corresponding to realistic experimental conditions.^15,17^ Note that the potential is depicted with respect to the Ag/AgCl scale (−0.197 V vs SHE) to facilitate comparison to experimental data. To get a first idea on the impact of an applied potential, the phase diagram of the pristine InP(100) surface reconstructions is considered; that is, chlorine and hydrogen adsorption are neglected. Still, this simplified model contains some important insights, showing that transitions between the different clean phases all would lie in a very narrow potential window (see Figure 7a). The transitions between the stable surface reconstructions all lie within about 0.1 V, with the In-rich mixed-dimer structure dominating for more cathodic potentials (below ∼−0.5 V) and the P-rich (2 × 2)-2D being dominant for more anodic potentials (above ∼−0.4 V).

These findings already indicate that α2(2 × 4), β2(2 × 4), and *c*(4 × 4) reconstructions are rather unlikely to be observed in an electrochemical environment, even under the assumption of negligible hydrogen and chlorine adsorption. On the other hand, when the full phase space is taken into account, the phase diagram in Figure 7b is obtained. At more cathodic potentials, hydrogen-terminated surfaces are observed, however, with different In/P content. In fact, with increasing (less cathodic) potential, the surfaces get more P-rich, resulting in transitions between surfaces with mixed In–P termination (based on (2 × 2)-mixed structures) to P-terminated (2 × 2)-based surfaces and (2 × 2)-2D-based phases. Interestingly, the hydrogen content on each of these surfaces decreases with increasing potential such that the hydrogen concentration on the surface does not uniformly decrease when the applied potential is increased. For instance, a transition from (2 × 2)-6H to (2 × 2)-2D-8H is predicted for increasing potential (at ∼−0.3 V), thus meaning an increase in hydrogen content, while with further increasing potential, hydrogen then desorbs again from the (2 × 2)-2D-type structure. At an applied potential of about 0.1 V, the (2 × 2)-2D-H-Cl phase with hydrogen and chlorine co-adsorption is stabilized, however, only in a tiny potential window. Hence, this means a rather sharp transition from hydrogen to chlorine adsorption.

For more anodic potentials, chlorine adsorption is dominant, quickly reaching the already rather distorted (2 × 2)-2D-8Cl surface. From the large phase space occupied by the In- and Cl-rich phases observed in Figure 5, it seems surprising that these structures are not present under electrochemical conditions. However, by connecting the applied voltage to the selected plots at fixed In chemical potentials, which are depicted in Figures 5a–c, this becomes more evident. The white pentagons in Figure 5a–c mark the phases that correspond to a particular applied voltage under the given electrochemical conditions for the depicted values of Δμ_In_. This means, for instance, that a potential of −0.25 V corresponds to a Δμ_In_ of −0.9 eV, with Δμ_Cl_ = −1.44 eV and Δμ_H_ = −0.01 eV (see Figure 5a), describing a point in the phase diagram where the (2 × 2)-2D-6H phase is found to be stable. By further examining the situations depicted in Figures 5a–c, it becomes clear that for increasing voltage, the electrochemical potential of In (and hydrogen) decreases, such that In-poorer (and H-poorer) surfaces become more favorable. Thus, when the potentials at which Cl adsorption becomes favorable are reached, there is at the same time a preference for P-terminated surfaces, such that In- and Cl-rich phases cannot be reached. As already mentioned in the general discussion on the CHE, this approach may introduce an uncertainty in the potential domain when comparing these results to experiment, meaning that the onset potential of transitions between different surface reconstructions may be shifted. Finally, it has to be noted that recent studies on hydrogen-terminated (2 × 2)-based phases demonstrated that doping can result in the stabilization of charged surfaces, depending on the Fermi level position.^36^ This indicates that applied potentials may also favor charged surfaces and/or affect the electronic structure.

**Figure 8:**
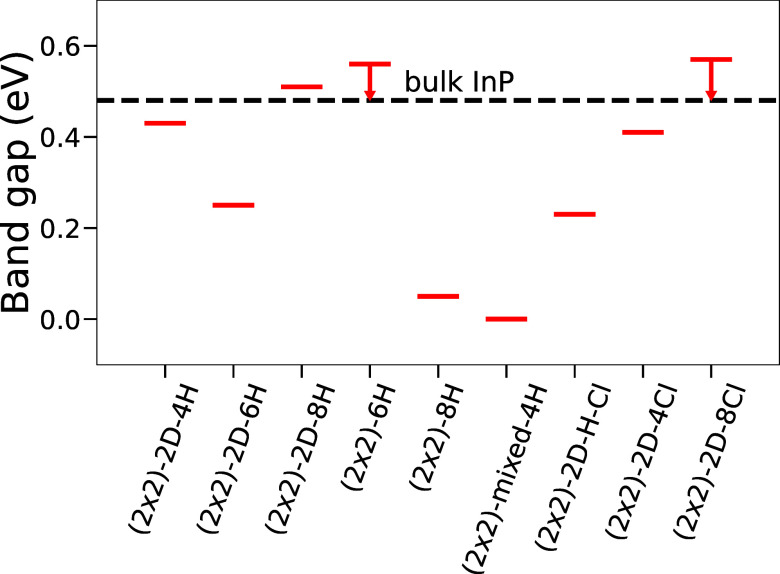
Calculated band gaps for the different surface reconstructions that are observed in Figure 7. The bulk band gap is depicted as a dashed line. The corresponding band structure plots are shown in Figure S2.

While the above results give valuable insights with respect to the different phases that are, or could in principle be, observed under experimental conditions, an open question is the impact of the adsorbates on the electronic properties. Surface reconstructions that create electronic states within the band gap of InP are detrimental for the use as a photoelectrode and should therefore be avoided. To investigate how the electronic structure is affected by the respective adsorbates, we determined the band structure for the different structures that are predicted to be stable under electrochemical conditions. Before discussing the results, it has to be noted that the applied PBE+D3 approach largely underestimates the band gap, a well-known issue for generalized gradient-based functionals, hence yielding a bulk band gap of ∼0.48 eV for InP. Furthermore, it has to be pointed out that due to the finite system size, band gaps larger than the bulk value can be observed.^37^ This finite size effect is, however, decreasing with an increasing slab size. Yet, for some of the surface reconstructions shown here (indicated by arrows in Figure 7), even a slab thickness of beyond 40 layers does not lead to full convergence. This is probably the reason why, for surface slabs of InP, band gaps closer to the experimental value (1.3 eV) than to the PBE-underestimated bulk band gap are frequently reported. The resulting band gaps are depicted in Figure 8 and compared to the bulk value, showing that for some phases the width of the band gap is largely affected. Band structure plots of the corresponding phases, showing midgap states or changes at the band edges, causing the observed changes in the band gap are depicted in Figure S2 in the SI. In fact, qualitatively the (2 × 2)-2D-based phases show band gaps close to the bulk value for (2 × 2)-2D-4H and the H-rich (2 × 2)-2D-8H phase, whereas (2 × 2)-2D-6H is affected by the presence of midgap states. A similar behavior is observed for the (2 × 2)-based phases where (2 × 2)-6H is observed to have a bulk-like gap, while the (2 × 2)-2D-8H becomes almost metallic. The (2 × 2)-2D-H-Cl phase also shows a decreased band gap, whereas for the Cl-rich phases, the band gap is again close to the bulk value, and no midgap states are observed. Hence, especially for the (2 × 2)-2D-8H, (2 × 2)-6H, and the Cl-rich (2 × 2)-2D-8Cl phases, no detrimental effects, such as charge-trapping in gap states, are expected. Consequently, these structures could be good starting points for (photo)electrochemical surface processing.

## Conclusion

By using DFT we were able to investigate the gas-phase adsorption of hydrogen and chlorine on InP(100), showing that coadsorption can only be observed for very small ranges of the chemical potential phase space. The novel type of phase diagram, which we introduce here, allows a quantification of phase stabilities with respect to competing phases, thus emphasizing that certain phases may be difficult to observe experimentally. The resulting phase diagrams clearly point to a dominant occurrence of phases featuring In–Cl bonds such as the InCl_3_-type surface. Surprisingly, these findings do not hold in an electrochemical environment. There, the differences in respective standard potentials and oxidation states of In, Cl, and H restrict the accessible part of the phase space such that In- and, at the same time, Cl-rich phases are not observed. The hydrogen coverage, on the other hand, does not simply decrease with more anodic potential but instead fluctuates with changing potential. This is because the more P-rich-based surface reconstructions are stabilized with increasing potential. Furthermore, a sharp transition from hydrogen to chlorine adsorption is observed, such that the hypothesis derived from experiment of H–Cl coadsorption seems rather unlikely. Finally, an investigation of the electronic band structure shows that the adsorption does not generally introduce midgap surface states in InP, which is a prerequisite for materials to be used as high-performance photoelectrode or processing of optoelectronic devices in electrochemical environments.

## Data Availability

The data underlying this study will be made openly available upon reasonable request to the authors.

## Supporting Information Available

Additional computational details and band structure plots (PDF)
