# Supplementary material for: The InP(100) Surface Phase Diagram: From the Gas Phase to the Electrochemical Environment
Source: ACS Appl Mater Interfaces. 2025 Jan 21;17(5):8601–9. doi: 10.1021/acsami.4c20370 (PMC11803560; doi:10.1021/acsami.4c20370)
Supplement: Supplementary file 1 — am4c20370_si_001.pdf [file am4c20370_si_001.pdf]

Supporting Information
The InP(100) Surface Phase Diagram: From the Gas-Phase to the Electrochemical Environment

Holger Euchner,* Vibhav Yadav, and Matthias M. May

Universität Tübingen, Institute of Physical and Theoretical Chemistry, Auf der Morgenstelle 15, 72076 Tübingen, Germany

E-mail: holger.euchner@uni-tuebingen.de

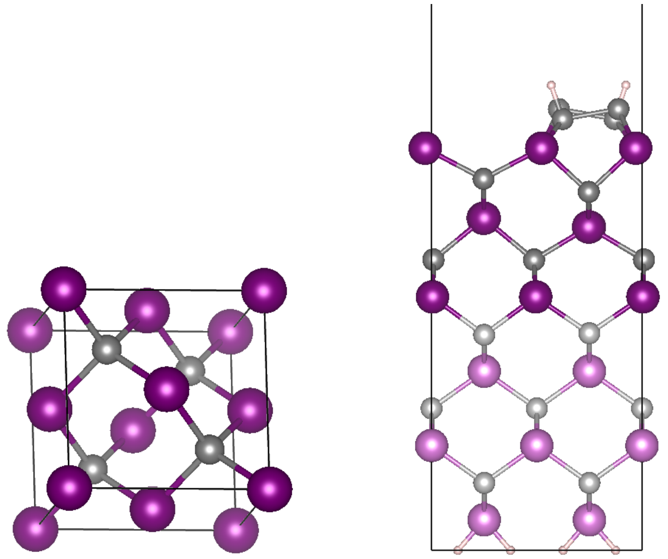

Figure S1: Bulk InP structure with In and P atoms in purple and grey (left). Schematic depiction of the computational setup for the InP(100) surface. The light grey (P) and light purple (In) atoms are kept fix during the calculations, while the positions of the In and P atoms in the top most layers (dark purple and dark grey) are optimized (right).

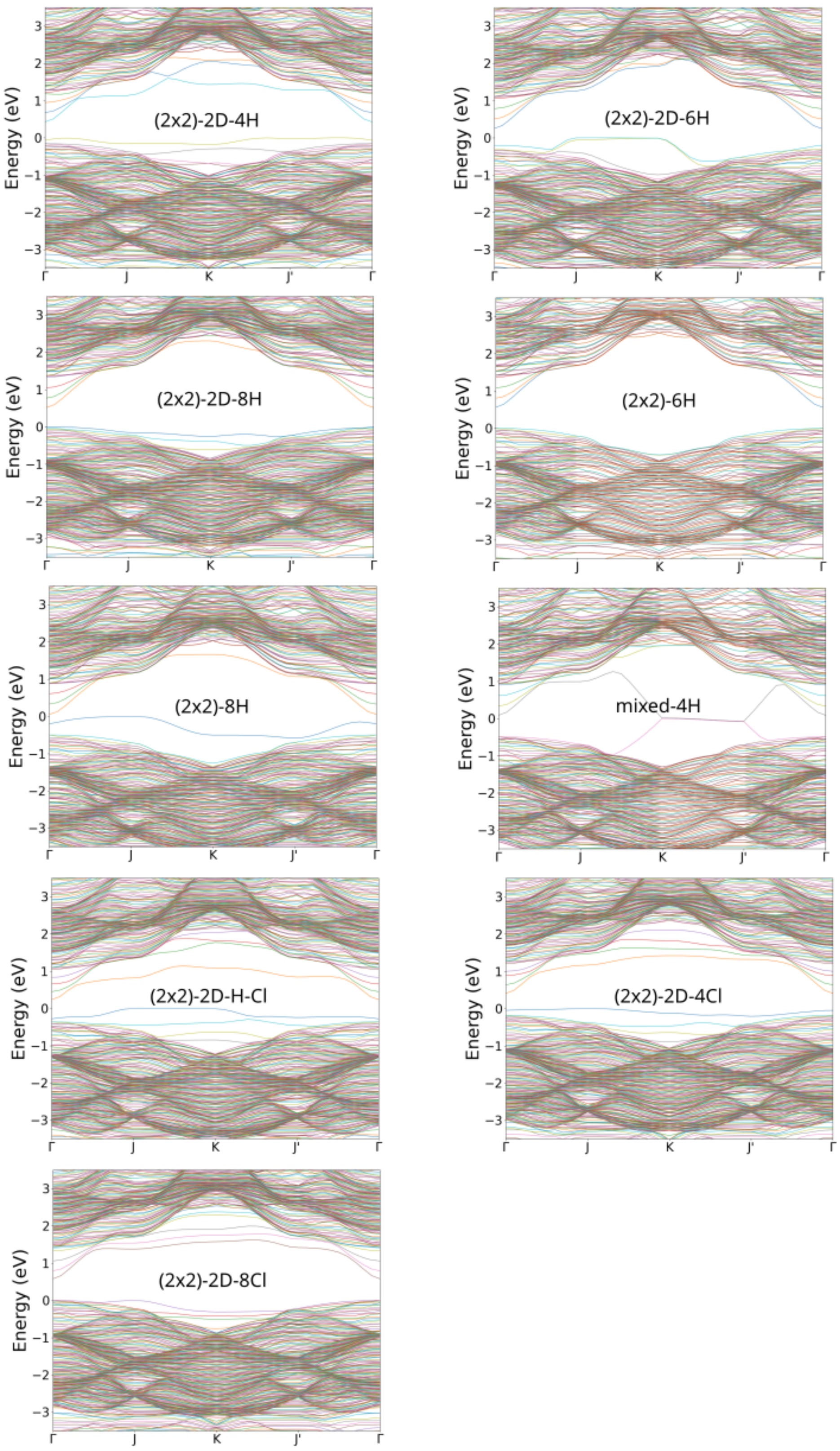

Figure S2: Band structure of the different stable phases that are observed under the given electrochemical conditions (see main text). The resulting band gaps for the different structures are given in Fig. 8 in the main manuscript.
